# Geller Syndrome: A Rare Cause of Persistent Hypokalemia During Pregnancy

**DOI:** 10.7759/cureus.26272

**Published:** 2022-06-24

**Authors:** Naif Hindosh, Rand Hindosh, Bolanle Dada, Swomya Bal

**Affiliations:** 1 Internal Medicine, St. Luke's University Health Network, Easton, USA; 2 Nephrology, St Luke's University Health Network, Easton, USA

**Keywords:** liddle syndrome, pre-eclampsia, monogenic disorders, refractory hypokalemia, recurrent hypokalemia, geller syndrome

## Abstract

Geller syndrome is a rare disease and part of Mendelian forms of hypertension. This syndrome is caused by a mutation in the mineralocorticoid receptor with a resultant gain of function. It is characterized by hypertension and hypokalemia, which is exacerbated by the effect of progesterone and thereby presenting during pregnancy. Our patient is a 22-year-old female diagnosed with preeclampsia who presented with hypokalemia, refractory to treatment toward the end of her third trimester. The patient's hypokalemia resolved once she delivered her infant. Genetic testing is available, which can confirm the diagnosis of Geller syndrome. The management is supportive therapy and requires close monitoring of the patient and her fetus. Delivery of the fetus results in the resolution of both hypertension and hypokalemia.

## Introduction

Monogenic forms of hypertension are defined as a set of disorders characterized by abnormal regulation of blood pressure by the kidney and adrenal gland [1]. Geller syndrome is a rare autosomal dominant disease attributed to a mutation in mineralocorticoid receptor (MR) resulting in a "gain of function", which presents with hypokalemia and hypertension during pregnancy, and usually resolves post-partum [2]. The diagnosis can be challenging due to the broad differential diagnosis associated with hypokalemia in the settings of hypertension. We present a case of persistent hypokalemia in a 22-year-old G1P0 female at 36 weeks gestation who was recently diagnosed with preeclampsia.

## Case presentation

22-year-old female G1P0A0 at 36 weeks gestation with recently diagnosed preeclampsia presented with low potassium level on an outpatient laboratory test. She had no history of hypertension or hypokalemia prior to her pregnancy. The patient reported intermittent episodes of headaches and visual disturbances during her third trimester. She had recently been admitted on three separate occasions for elevated blood pressure and was noted to have low potassium levels during each admission. The patient denied any gastrointestinal symptoms such as vomiting or diarrhea that could explain the persistent hypokalemia. She had no urinary complaints. She also denied alcohol consumption or the use of any herbal supplements such as licorice. Other than prenatal vitamins, the patient did not take additional medications. She was not aware of a family history of hypertension and hypokalemia. Vital signs on admission showed a temperature of 98.2F, heart rate of 88 per minute, respiratory rate of 14 per minute, and blood pressure of 144/90mmHg. Physical examination was notable for trace bilateral leg swelling and gravid uterus consistent with 36 weeks gestation, otherwise unremarkable exam. Initial laboratory studies are shown in Table 1. 

**Table 1:** Laboratory values BUN - blood urea nitrogen; eGFR - estimated glomerular filtration rate; WBC - white blood count; AST - aspartate aminotransferase; ALT - alanine aminotransferase; ALP - alkaline phosphatase

Test	Value	Reference range
Sodium	140	135-145 mmol/L
Potassium	2.5	3.5-5 mmol/L
Chloride	104	96-108 mmol/L
Bicarbonate	23	22-33 mmol/L
Magnesium	1.3	1.6-2.6 mg/dL
BUN	6	6-20 mg/dL
Creatinine	0.63	0.4-1.1 mg/dL
eGFR	101	ml/min/1.73sq m
Hemoglobin	9.5	11.5-15.4 g/dL
WBC	11.5	4.31-10.16 thousand/uL
Platelets	207	149-390 thousand/uL
ALT	17	12-78 U/L
AST	20	5-45 U/L
ALP	111	46-116 U/L
Total protein	6.8	6.4-8.2 g/dL
Albumin	2.8	3.5-5 g/dL

The patient was started on intravenous potassium and magnesium supplements; however, potassium levels continued to decline to a range of 2.5-2.7 mmol/L after discontinuing the potassium supplements and remained low despite normalization of magnesium level. Further workup revealed normal thyroid-stimulating hormone (TSH) and cortisol levels, with a renin level of 1.597 ng/mL/hr (0.167-5.380 ng/mL/hr) and suppressed aldosterone level (below 1 ng/dL). Urinary potassium was 38 mmol/L which was suggestive of a component of a renal potassium wasting as an etiology for her low potassium levels.

Preeclampsia worsened during this hospitalization requiring induction of labor with the fetus eventually delivered via cesarean section. Within 48 hours following delivery, blood pressure normalized, and potassium levels started to improve as well and eventually normalized when rechecked within 10 days postpartum, even though potassium supplements had been discontinued.

## Discussion

Since its description by Geller et al. in 2000, less than 10 cases of Geller syndrome have been reported, including our case, to the best of our knowledge [3-6]. Despite its rarity, Geller syndrome should be a condition that physicians are aware of, during pregnancy, because of the agonist effect of progesterone on the mutated MR, leading to severe hypokalemia and high blood pressure [4]. The mutation is present on chromosome 4q31 and results from leucine substitution for serine at amino acid 810 [7]. Renin and aldosterone levels are usually suppressed in this syndrome [1]. There was also a suspicion that the patient's presentation could be related to Liddle syndrome; however, the development of hypertension and hypokalemia during pregnancy and resolution thereafter inclined more toward the diagnosis of Geller syndrome. Reasons for not considering the other conditions that can present with hypertension and refractory hypokalemia as the cause of our patient's presentation are listed in Table 2 [1,4,8].

**Table 2:** Other conditions that can present with refractory hypokalemia and hypertension

Condition	Reason of exclusion
Liddle syndrome	Described above
Apparent excess of mineralocorticoid (AME)	Presents during early childhood
Primary/familial hyperaldosteronism	Presents with high aldosterone level
Cushing syndrome	The patient lacked the classic sign and symptoms to support this diagnosis
Renin secreting tumor/renal artery stenosis	Present with high renin and aldosterone levels
Congenital adrenal hyperplasia	Presents during infancy with atypical sexual development; cortisol level is usually low

Gitelman and Bartter syndrome can cause refractory hypokalemia as well; however, patients who are diagnosed with these syndromes have normal or low blood pressure with elevated renin and aldosterone levels [8,9]. Genetic testing remains the gold standard for confirmation of the diagnosis [1]; unfortunately, this was unavailable for our patient. Treatment of Geller syndrome is usually supportive and is focused on close observation of the fetus and mother while managing hypertension and hypokalemia, which usually resolve after delivery of the fetus. Some studies have shown that patients responded well to amiloride, while spironolactone has been noted to worsen blood pressure by activating the mutated MR, therefore contraindicated in Geller syndrome [3,10].

## Conclusions

Early recognition and awareness of Geller syndrome are crucial to avoid serious complications for the patient and fetus from hypokalemia and hypertension. Amiloride has shown effectiveness in some cases, whereas spironolactone is contraindicated as it may exacerbate the condition. Genetic testing can confirm the diagnosis. It is important to educate the patient about the high chances of recurrence during future pregnancies and the need for close monitoring of the blood pressure and potassium levels with the primary care provider.
